# Phenotypically Anchored mRNA and miRNA Expression Profiling in Zebrafish Reveals Flame Retardant Chemical Toxicity Networks

**DOI:** 10.3389/fcell.2021.663032

**Published:** 2021-04-09

**Authors:** Subham Dasgupta, Cheryl L. Dunham, Lisa Truong, Michael T. Simonich, Christopher M. Sullivan, Robyn L. Tanguay

**Affiliations:** ^1^The Sinnhuber Aquatic Research Laboratory, Department of Environmental and Molecular Toxicology, Oregon State University, Corvallis, OR, United States; ^2^Center for Genome Research and Computing, Oregon State University, Corvallis, OR, United States

**Keywords:** flame retardants (additives, reactives), micro-RNA (miRNA), network analysis, transcription factors (TFs), neurodevelopment, zebrafish, mRNA, gene expression

## Abstract

The ubiquitous use of flame retardant chemicals (FRCs) in the manufacture of many consumer products leads to inevitable environmental releases and human exposures. Studying toxic effects of FRCs as a group is challenging since they widely differ in physicochemical properties. We previously used zebrafish as a model to screen 61 representative FRCs and showed that many induced behavioral and teratogenic effects, with aryl phosphates identified as the most active. In this study, we selected 10 FRCs belonging to diverse physicochemical classes and zebrafish toxicity profiles to identify the gene expression responses following exposures. For each FRC, we executed paired mRNA-micro-RNA (miR) sequencing, which enabled us to study mRNA expression patterns and investigate the role of miRs as posttranscriptional regulators of gene expression. We found widespread disruption of mRNA and miR expression across several FRCs. Neurodevelopment was a key disrupted biological process across multiple FRCs and was corroborated by behavioral deficits. Several mRNAs (e.g., *osbpl2a*) and miRs (e.g., mir-125b-5p), showed differential expression common to multiple FRCs (10 and 7 respectively). These common miRs were also predicted to regulate a network of differentially expressed genes with diverse functions, including apoptosis, neurodevelopment, lipid regulation and inflammation. Commonly disrupted transcription factors (TFs) such as retinoic acid receptor, retinoid X receptor, and vitamin D regulator were predicted to regulate a wide network of differentially expressed mRNAs across a majority of the FRCs. Many of the differential mRNA-TF and mRNA-miR pairs were predicted to play important roles in development as well as cancer signaling. Specific comparisons between TBBPA and its derivative TBBPA-DBPE showed contrasting gene expression patterns that corroborated with their phenotypic profiles. The newer generation FRCs such as IPP and TCEP produced distinct gene expression changes compared to the legacy FRC BDE-47. Our study is the first to establish a mRNA-miR-TF regulatory network across a large group of structurally diverse FRCs and diverse phenotypic responses. The purpose was to discover common and unique biological targets that will help us understand mechanisms of action for these important chemicals and establish this approach as an important tool for better understanding toxic effects of environmental contaminants.

## Introduction

Flame retardant chemicals (FRCs) have been ubiquitous in furniture, electronics, carpets, textiles, automotive products and children’s clothing for decades. Throughout the 1980s and 1990s, polybrominated diphenyl ethers (PBDEs) were the predominant flame retardants, but these were phased out in 2005 due to concerns of environmental persistence, bioaccumulation and toxicity. Replacement with organophosphate flame retardant chemicals (OPFRCs) has been common since 2005 (Stapleton et al., 2012). OPFRCs may be halogenated or non-halogenated alkyl or aryl phosphates and are often additives, not chemically bound to the items they protect. FRCs are widely detected in household dust, personal vehicles, indoor air, and aquatic environments due to leaching (Stapleton et al., 2009; Hoffman et al., 2015b; Reddam et al., 2020). Furthermore, exposure assessment studies have detected measurable levels of these chemicals in breast milk, urine and blood from human samples (Hoffman et al., 2015a, b). Exposures to both PBDEs and OPFRCs are associated with neurological and reproductive deficits within human populations (Herbstman and Mall, 2014; Castorina et al., 2017; Doherty et al., 2019). Developmental health is of particular concern, since development is regulated by a complex interplay of biological processes that regulate cell migration, differentiation and organogenesis and hence are sensitive to the effects of external stressors (Dasgupta et al., 2018). *In utero* exposure to FRCs is not uncommon as these chemicals are measured in placental or cord blood samples from the developing fetus (Kawashiro et al., 2008; Ding et al., 2016). Over the last two decades, several studies have explored toxic effects of various classes of FRCs in a wide range of *in vivo* and *in vitro* developmental models using high throughput screening, transcriptomics, metabolomics, epigenetics and reverse genetics. Collectively, evidence indicates that FRC exposure disrupts morphogenesis, neurodevelopment, immunodevelopment, muscle development, metabolism and development of various organs such as heart and liver (Du et al., 2019). Discovering the toxic mechanisms of FRCs as a group is challenging since they have widely varied physicochemical properties. Toxicity comparison among existing laboratory-based studies is somewhat limited by variation in exposure paradigms, targeted biological processes and life stages.

We previously used high-throughput screening in zebrafish with statistical modeling to identify developmental toxicity of FRCs based on their physicochemical classifications and bioactivity profiles (Noyes et al., 2015; Hagstrom et al., 2019; Truong et al., 2020). Recently, we screened a comprehensive library of 61 FRCs for morphological and neurobehavioral endpoints and used a point-of-departure approach to show that FRCs from several classes elicited developmental toxicity (Truong et al., 2020). In that study, we built a robust classification model for FRCs based on phenotypes and physicochemical properties and also showed that triphenyl OPFRCs such as triphenyl phosphate (TPP) and isopropylated triphenyl phosphates (IPPs) were the most bioactive. Comparisons with other model systems showed that many of the bioactive FRCs in our study were toxic in mammalian *in vivo* and *in vitro* systems (Truong et al., 2020). These data revealed the need for a comparative molecular assessment of different FRC classes to understand their unique and common biological targets and how specific biological processes are associated with phenotypes.

Here we investigated the mRNA and miR expression changes following exposure to 10 selected FRCs representing different physicochemical classes and zebrafish toxicity response profiles, ranging from non-responders to high responders (Truong et al., 2020). miRs can act as important post-transcriptional regulators of biological processes in response to an array of metals, dioxins, microcystins, phenols, PM_2_._5_ and are associated with teratogenic effects, apoptosis, hepatotoxicity, metabolic disruptions, carcinogenesis, neurotoxicity and oxidative stress (Balasubramanian et al., 2020). There is evidence that specific miRs are involved in developmental and neurodevelopmental toxicity in zebrafish following exposures to several environmental contaminants such as 2,3,7,8-Tetrachlorodibenzodioxin (TCDD) (Jenny et al., 2012) and atrazine (Wirbisky et al., 2016). We report the first FRC-associated paired mRNA and miR sequencing study in developmental zebrafish. Ten representative FRCs with zebrafish exposures from 6 to 48 h post fertilization (hpf) were chosen to quantify early gene expression changes that later (120 hpf) manifest as phenotypes. We anchored our mRNA/miR expression data to the phenotypes and identified several differentially expressed genes, mRNA-miR interactions, and transcription factors (TFs) linked to the toxicity of OPFRCs. Finally, we share an FRC-gene expression database to hopefully inspire and facilitate additional functional studies.

## Materials and Methods

### Chemicals

**FIGURE 1:**
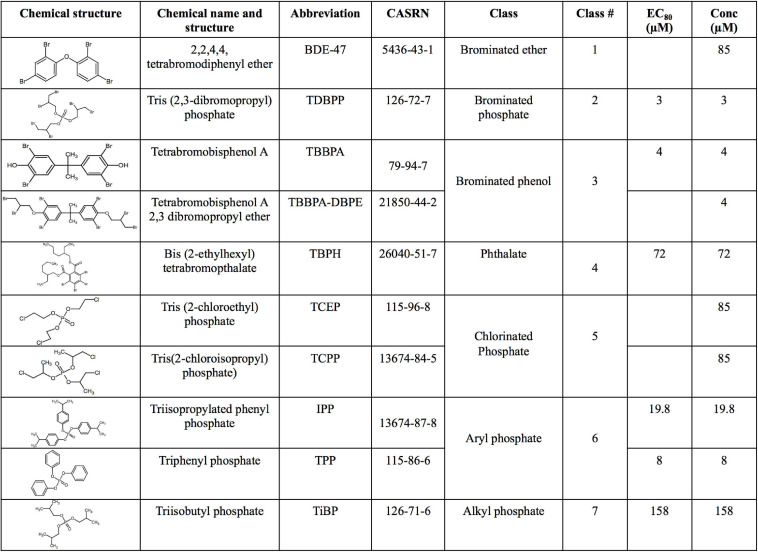
Chemical name, structure, abbreviation, CAS number, physicochemical classes, EC_80_ s and exposure concentrations (“Conc”) of flame retardant chemicals (FRCs) used in this study. “Conc” represents concentration used for exposures in the study. For FRCs, EC_80_s [exposure concentrations demonstrating 80% morphological effects based on Truong et al. (2020)] were used as exposure concentrations, except BDE-47, TCEP and TCPP where limit concentration (85 μM) or TBBPA-DBPE where a TBBPA-matched concentration (4 μM) was used.

The US Environmental Protection Agency, as part of the grant number #83579601, generously provided the 10 FRCs (see Figure 1) at 20 mM in 100% DMSO. The chemicals had >98% purity and were provided in a 96 well plate and stored at −80° prior to exposing zebrafish embryos.

### Selection of FRCs and Their Exposure Concentrations

**FIGURE 2:**
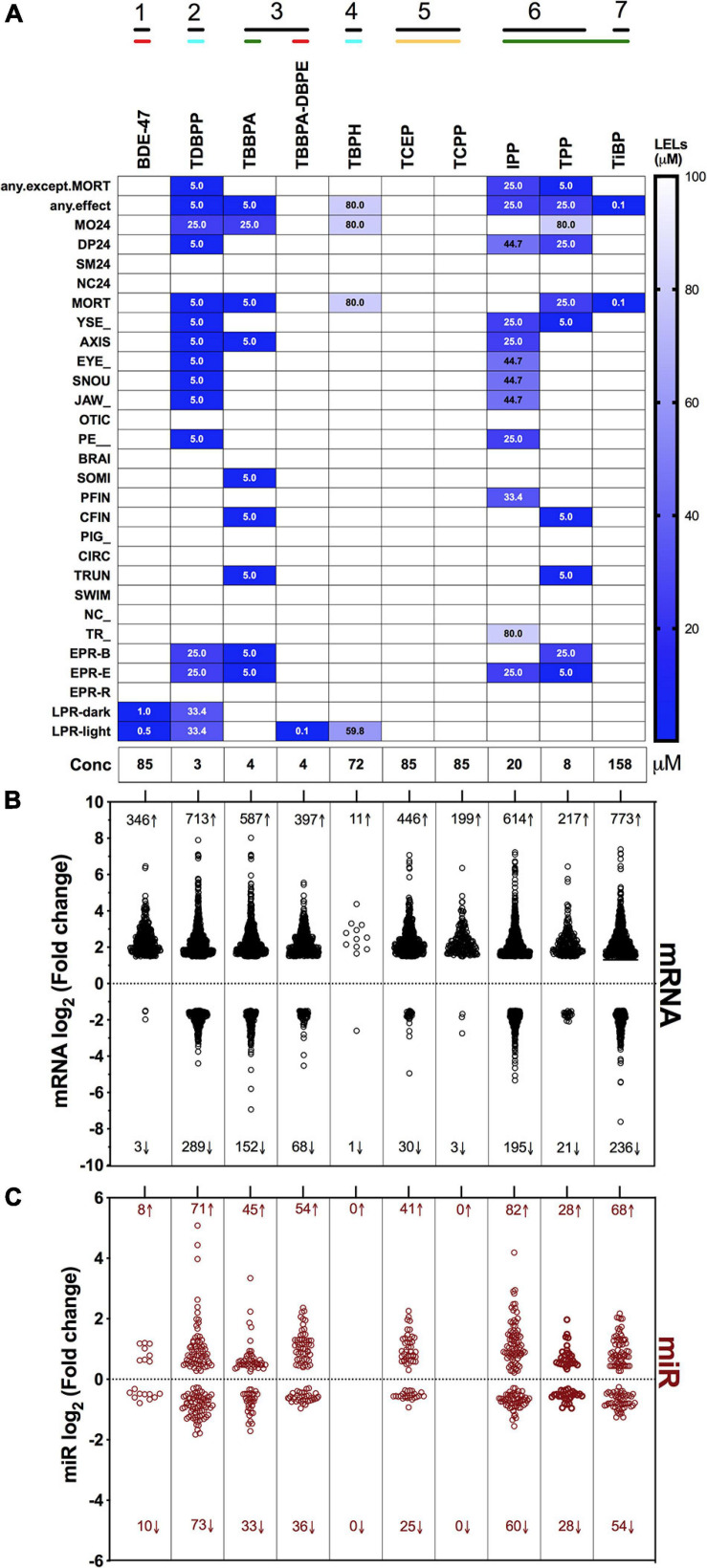
Phenotypic screening, mRNA sequencing and miR sequencing of 10 FRCs. **(A)** Phenotypic screening with lowest effect level (LEL) of 18 morphological endpoints and four behavioral endpoints; details about these endpoints are included in Supplementary Table 1. Data based on Truong et al. (2020). Exposure concentrations for this study are represented as “Conc.” Black lines with numbers indicate FRC classes based on Figure 1. Colored lines indicate phenotypic response groups: 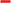 only behavior response; 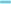 morphology + behavior response; 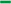 only morphology response and 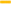 no response. Embryonic photomotor response is included within morphology response and behavior response indicates only larval photomotor response. Numbers within cells represent LELs for each FRC/endpoint combination. **(B)** Log_2_ fold changes of all increased and decreased differentially expressed mRNAs across FRCs. Cutoff was log_2_ fold change ≥1.5 and *p* ≤ 0.05. Numbers represent number of genes with increased (↑) or decreased (↓) mRNA levels. **(C)** Log_2_ fold changes of increased and decreased miR levels across different FRCs. Cutoff was *p* ≤ 0.05. Numbers represent number of miRs with increased (↑) or decreased (↓) levels.

Our objective was to discover the gene expression changes produced by exposure to FRCs belonging to different physicochemical classes and zebrafish toxicity profiles. We chose the 10 FRCs based on lowest effect level (LEL) values within our 18 morphological and 5 behavioral endpoints from our previous study [Truong et al. (2020), also see Supplementary Table 1.1)]. These FRCs belonged to different chemical classes (Figure 1) and phenotypic profiles (Figure 2A), producing both morphological and behavioral phenotypes (TDBPP, TBBPA, TBPH, IPP, TPP), only morphological phenotypes (TiBP), only behavioral phenotypes (BDE-47, TBBPA-DBPE) and no observed phenotypes (TCEP, TCPP) (Figure 2A). Since none of the FRCs elicited an abnormal embryonic photomotor response (EPR; 24 hpf) phenotype without also manifesting a later morphological phenotype, our use of “behavioral phenotypes” here typically pertains to the later (120 hpf) larval photomotor response (LPR). Based on data from Truong et al. (2020), we calculated 120 hpf EC_80_-concentrations that caused 80% of the embryos to be adversely affected in their morphology by 120 hpf and performed paired mRNA and miR sequencing at these concentrations to quantify gene expression changes at near-maximal responses. For TBBPA-DBPE, we matched the exposure concentration (4 μM) to its parent FRC TBBPA. For FRCs with no morphological phenotypes (TCPP, BDE-47, and TCEP), a limit concentration of 85 μM was used.

### Zebrafish Husbandry

Tropical 5D wild type zebrafish were housed at Oregon State University’s Sinnhuber Aquatic Research Laboratory (SARL, Corvallis, OR, United States) in densities of 500 fish per 100-gallon tank according to the Institutional Animal Care and Use Committee protocols. Fish were maintained at 28°C on a 14:10 h light/dark cycle in recirculating filtered water, supplemented with Instant Ocean salts. Adult, larval and juvenile fish were fed with size appropriate GEMMA Micro food 2–3 times a day (Gaulke et al., 2016). Spawning funnels were placed in the tanks at night, and the following morning, embryos were collected and staged (Kimmel et al., 1995; Westerfield, 2007). Mass collection of embryos from these tanks allows for multiple individual fish and male-female pairs contributing to embryos for an experimental setup and provides for sufficient genetic diversity. Embryos were maintained in embryo medium (EM) in an incubator at 28°C until further processing. EM consisted of 15 mM NaCl, 0.5 mM KCl, 1 mM MgSO_4_, 0.15 mM KH_2_PO_4_, 0.05 mM Na_2_HPO_4_, and 0.7 mM NaHCO_3_ (Westerfield, 2007).

### Chemical Exposures

Embryos were dechorionated at 4 hpf, sorted based on their stage (Kimmel et al., 1995) and shield stage embryos (6 hpf) were exposed in 96 well plates (one embryo per well in 100 μL test solution) as described in Truong et al. (2020). Details of exposure concentrations and observed phenotypes are included in Figure 2A and Supplementary Table 1.1. A vehicle control of 0.64% DMSO was used because previous studies from our and other labs have shown no confounding physiological effects at 0.64%. The embryos (*N* = 48) were statically exposed from 6 to 48 hpf at 28°C in the dark.

### Total RNA Extraction and mRNA/miRNA Sequencing

We collected embryos at 48 hpf to study the transcriptomic dynamics that precede morphological and/or behavioral phenotypes at 120 hpf. This exposure paradigm provides a snapshot of the developmental transcriptome in absence of any phenotypes at 48 hpf, but largely drives phenotypic outcomes at 120 hpf. Four biological replicates were created by pooling eight embryos per replicate from individual wells which were placed into an Eppendorf Safe-lock Tube and excess solution removed. 0.5 mM zirconium oxide beads were added along with 500 μL of RNAzol (Molecular Research Center, Inc.) and the tubes were immediately placed into a bullet blender (Next Advance), using settings recommended by the manufacturer. The RNA was purified using the Direct-zol MiniPrep kit (Zymo Research), including an optional DNase-1 digestion treatment for 15 min. RNA integrity (RIN) was assessed using an Agilent Bioanalyzer (Santa Clara, CA, United States), and RNA samples with RIN values >8 were processed for library preparation and sequencing at the Oregon State University Center for Genome Research and Biocomputing. Total RNA was used as input for both mRNA and miRNA sequencing. For mRNA sequencing, mRNA was poly A selected, libraries were prepared with the PrepX mRNA and Illumina sequencing workflow (Wafergen Biosystems). For miRNA seq, the Illumina TruSeq Small RNA library kit was used to generate small RNA libraries from total RNA. For sequencing, an Illumina HiSeq 3000 sequencer (Illumina, San Diego) was used for mRNA and small RNA single-end sequencing at 100 base pairs; for miR sequencing, the insert sizes were upto 50 bp. Bioinformatics analysis of sequencing data was performed on an R platform. Briefly, reads were evaluated by FastQC v0.11.3 (Andrews, 2015) to detect major sequencing problems, and then trimmed for quality control with Skewer v0.2.2 (Jiang et al., 2014). RNA-seq alignment and quantification proceeded with Bowtie2 v2.2.3 (Langmead et al., 2009; Langmead and Salzberg, 2012) being used to build HISAT2 (Kim et al., 2015, 2019) genome index files from the Genome Reference Consortium Zebrafish Build 10 (GRCz10) genome. Gene counts were estimated using HTSeq v0.9.1 (Anders et al., 2015) with the GRCz10 Ensembl 91 GTF annotation. For miR identification and quantification, a combination of miRDeep2 v2.0.0.8, miRBase release 22 and Bowtie v1.2.1.1 were used. Differential expression between experimental and control samples was determined with functions from the Bioconductor package, edgeR; mRNAs with a log_2_ fold change ≥1.5 and Benjamini-Hochberg (BH) adjusted *p* ≤ 0.05 were considered differentially expressed while an adjusted *p* ≤ 0.05 was applied to miRs without any fold change cutoffs. Heatmap clustering of differentially expressed genes were generated in R based on their log_2_ fold changes using the ggplot2 package. Expression data for statistically significant and cutoff-applied mRNA and miR are included within Supplementary Tables 1.2, 1.3. Detailed methods for mRNA and miR sequencing, including codes, are available within the following link: https://github.com/Tanguay-Lab/Manuscripts/tree/main/Dasgupta_et._al._(2021)_Front_Cell_Dev_Biol.

### Co-regulatory Analysis and Data Visualization for miR, Genes and Transcription Factors

For integrative analysis of TFs, miRs and mRNA targets, we first identified human orthologs for differentially expressed genes using The Bioinformatics Resource Manager (BRM) (Brown et al., 2019). Next, we imported human gene symbols, miRs and their directionality of fold changes (+1 for upregulation, −1 for downregulation) for each FRC into TFmiR^1^, a freely available web platform (Hamed et al., 2015). TFmiR uses an array of databases such as miRTarBase, TransmiR, ChipBase, TRANSFAC to generate mRNA-TFs, TF-miR, gene-miR and miR-miR interactions and co-regulations from input data and can categorize co-regulations as “experimental” (experimentally validated co-regulation through prior functional studies) or “predicted” (computationally predicted co-regulations) distinctly. We analyzed data using the following configuration within TFmiR: *p* threshold-0.05; related disease: no disease and evidence: both experimental and predicted. For gene-miR interactions, we only considered anti-correlated pairs (e.g., + 1 for gene and −1 for miR) for all downstream interpretations. For visualization and discussion of interactions in the manuscript, we only used “experimental” outputs; therefore, within the manuscript, “predicted to regulate” refers to co-regulations with evidence of experimental validation from previous studies based on TFmiR. Lists of interactions between mRNA, TFs and miRs based on the TFmiR outputs were imported into Cytoscape^2^ or GraphPad Prism 9 (San Diego, CA, United States) to generate networks and heatmaps of FRC-mRNA-miRs, FRC-mRNA-TFs or mRNA-TF-miRs for each FRC. It is to be noted that since all co-regulation analysis was done based on human orthologs, the discussion on these networks is presented with human gene symbols. All TFmiR outputs are included within Supplementary Table 2.

### Gene Ontology Analysis

To understand the biological consequences of chemical exposure, we performed GO term enrichment using GeneGo MetaCore version-19.3 build-69800 from Clarivate Analytics, as described in Garcia et al. (2018). For gene expression data, we imported BRM-generated human gene symbols and their fold changes into MetaCore and performed GO process analysis. For miR data, we sought to assess the biological processes impacted by target genes of miR. Therefore, for each FRC, we imported the TFmiR-generated target gene list (here using both experimentally validated and computationally predicted data) for gene-miR interactions and performed GO process analysis. GO terms with a false discovery rate (FDR) adjusted *p* ≤ 0.05 were considered significant and data was represented as heatmaps (GraphPad Prism 9, San Diego, CA, United States). All GO outputs are included within Supplementary Table 3.

## Results

### Changes in mRNA and miR Expression Across FRCs

Overall, there was a general proclivity toward increased gene expression compared to reduced gene expression (Figure 2B). Consistent with their phenotypic effects, TDBPP, TBBPA, IPP and TPP elicited the most extensive changes in mRNA expression. For example, IPP exposures resulted in 614 increased and 195 decreased mRNAs. FRCs with minimal (BDE-47, TBBPA-DBPE, TiBP) or no (TCEP, TCPP) phenotypic effects also induced significant changes in mRNA expression. Notably, BDE-47 and TCPP, which did not produce morphological phenotypes, elicited extensive increase, but limited decrease in expression levels of mRNAs. Conversely, for TBPH, an FRC with substantial phenotypic effects, transcriptomic changes were minimal. The extent of miR expression changes generally mirrored gene-level disruptions with the exception of BDE-47, TBPH and TCEP where we measured minimal or no miR expression changes (Figure 2C).

### Gene Ontology Analysis

**FIGURE 3:**
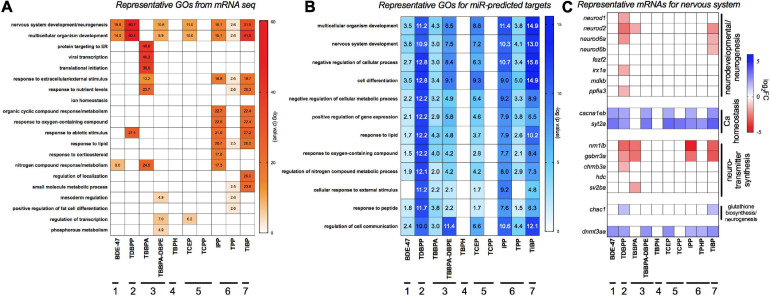
Major gene ontology (GO) processes across FRCs based on **(A)** differentially expressed mRNAs and **(B)** target mRNAs of differentially expressed miRs. GO was analyzed using human orthologs within Metacore. For panel **(B)**, differentially expressed miRs were imported into TFmiR and both experimentally validated, and computationally predicted gene targets were imported into Metacore for GO analysis. Data is represented as -log (FDR *p* value) for each term; a value of ∼1.3 represents FDR *p* threshold of 0.05. Numbers within cells represent the significant -log (FDR *p* value). **(C)** Expression of representative mRNAs known to regulate nervous system development and neurotransmitter activity. Horizontal lines with numbers (1–7) represent FRC class based on Figure 1.

Figure 3A shows gene ontology (GO) assessments of differentially expressed genes. We also studied GO analysis of mRNA targets that were predicted (both experimentally and computationally) to be anti-correlated to differentially expressed miRs in our data (Figure 3B) since miRs can negatively regulate gene expression. Multicellular development and nervous system were the most commonly affected processes for both miRs and mRNAs across a majority of the FRCs, with TDBPP, IPP and TiBP eliciting the strongest responses. The only exception was TBBPA, where protein-targeting and translational processes were the major disrupted processes. Other affected processes included lipid response, metabolic processes, mesoderm formation and transcriptional regulation. TBPH and TCPP did not show any statistically significant gene enrichment process. Figure 3C shows representative genes important for nervous system development. We showed that, for many FRCs (except TBBPA), genes that regulated neurotransmitter synthesis and neuronal development were repressed while genes that drive calcium homeostasis, important for neuronal function, were overexpressed. It is particularly noteworthy that expression of *dnmt3aa*, a neuronal methylation recruiter, was elevated by several FRCs.

### Clustering and Expression of Top Differentially Expressed Genes or miRs Across FRCs

**FIGURE 4:**
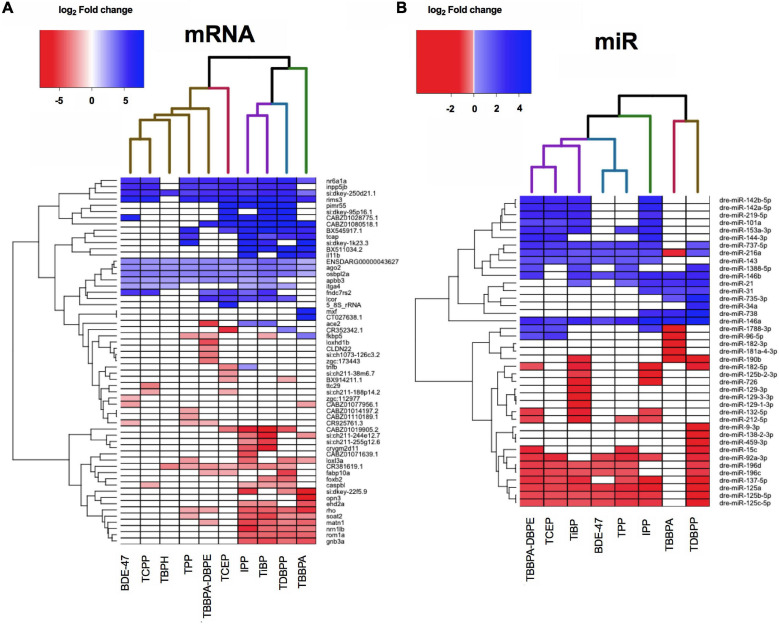
Heatmap representing log_2_ fold changes of **(A)** top mRNAs and **(B)** top miRs across FRCs. Up to 5 genes with highest increase and decrease in mRNA or miR expression levels were selected and their fold changes were plotted for all FRCs. Each column dendrogram color represents a cluster.

We next evaluated if FRCs belonging to the same class (e.g., aryl phosphates, chlorinated phosphates, phthalates) clustered together based on expression data, and identified specific mRNAs and miRs with the strongest expression changes. We chose the five most increased and five most decreased transcripts for each FRC and plotted their expression across all FRCs. This identified 59 mRNAs and 41 miRs after accounting for unique targets and FRCs with <5 increased or decreased transcripts. None of the specific classes of FRCs clustered together for either mRNA (Figure 4A and Supplementary Figure 1A) or miRs (Figure 4B and Supplementary Figure 1B). Based on expression of the most altered mRNAs, IPP, TDBPP and TBBPA (all with morphological phenotypes), were close neighbors, although IPP also clustered with TiBP–an FRC with no observed morphological defects (Figure 4A). BDE-47, a penta-BDE that has been phased out due to toxicity, clustered closely with newer generation aryl and chlorinated phosphates such as TPP and TCEP. Based on individual transcripts, at least 9 of the 10 FRCs shared increased transcripts such as *nr6a1a* (a transcription factor), *rims3* (regulates synaptic transmission), *ago2* (binds miRs) and *osbpl2a* (regulates lipid transport). Common reduced transcripts were scarcer, with *rho* (regulates photoreceptor activity) and *matn1* (regulates cartilage development) being differentially expressed for 6–7 FRCs. mRNA levels of several unannotated transcripts such as *si:dkey-250d21.1* were differentially altered by multiple FRCs, highlighting the need to identify the functions of these genes. Based on miR data (Figure 4B), three FRCs that produced substantial changes in miR expression but minimal or no phenotypes-TBBPA-DBPE, TCEP and TiBP-clustered together. The expression of a number of common miRs were significantly decreased across multiple FRCs (Figure 4B); top increased miRs included mir-142b-5p, mir-727-5p and decreased miRs included mir-125c-5p and mir-137-5p; their roles in gene expression are discussed in subsequent sections.

### mRNA–miR Interactions

**FIGURE 5:**
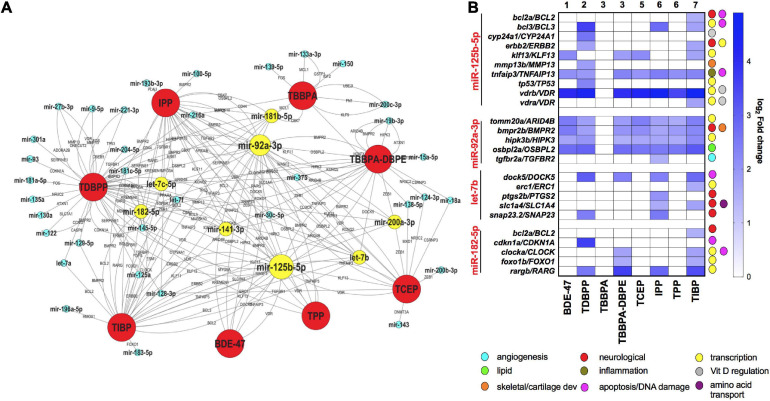
mRNA-miR interactions across FRCs based on experimentally validated predictions. **(A)** FRC-mRNA-miR co-regulatory network based on TFmiR gene-miR interactions. Only mRNA-miR pairs with reverse expression directions were considered for the network. 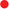 FRCs, 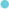 miRs, 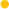 miRs that were differentially expressed across 4 or more FRCs, with larger nodes denoting higher number of FRCs. mRNAs are represented within the connected lines. **(B)** Selected miRs that were decreased across multiple FRCs, with fold changes and major functions (based on GO analysis) of their anti-correlated mRNA targets in specific FRCs. Numbers represent FRC classes based on Figure 1. TBPH and TCPP not represented since there were no miR disruptions.

To explore the network of miRNAs with their anti-correlated target mRNAs across different FRCs, we constructed an FRC–mRNA–miR network, based on experimental predictions (Figure 5A). Several miRs that were differentially expressed across multiple FRCs, such as mir-92a-3p and mir-125-5p (larger nodes), were also predicted to regulate a large network of differential mRNAs. These target mRNAs encompassed a plethora of major biological functions, the most frequent ones being transcription, apoptosis and nervous system development (Figure 5B). Levels of several miR-mRNA combinations, such as mir-125b-5p/vitamin D regulator (*VDR*) and mir-92a-3p/*HIPK3* were altered across all FRCs showing miR disruption (except TBBPA), suggesting that these interactions may drive a common mechanistic landscape for FRCs.

### Comparison of TBBPA-DBPE and TCEP for Neurodevelopmental Toxicity Biomarkers

**FIGURE 6:**
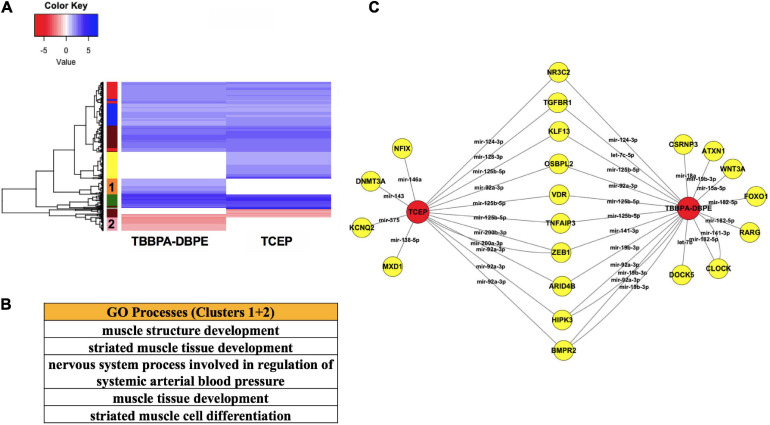
Comparison of TBBPA-DBPE and TCEP for neurotoxic effects. TBBPA-DBPE exposures showed only LPR phenotypes while TCEP showed no phenotype. **(A)** Heatmap representing mRNA expression for the two FRCs; colored bars on the rows represent gene clusters. **(B)** GO processes for unique differential mRNAs with increased (Cluster 1) and decreased (Cluster 2) in the TBBPA-DBPE exposures. **(C)** mRNA-miR network for the two FRCs. 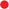 FRC, 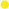 genes. miRs are represented within connected lines.

To better understand the molecular basis for neurobehavioral effects, we compared mRNA-miR networks between TBBPA-DBPE (no morphological defects but LPR abnormalities at 120 hpf) and TCEP (no phenotype). Gene ontology analysis of transcripts that were differentially altered uniquely by TBBPA-DBPE showed muscle development as the major process disrupted (Figures 6A,B). Several miR-mRNA pairs were significantly altered exclusively in TBBPA-DBPE, including mir-15a-5p/WNT3A, a Wnt signaling gene known as a crucial regulator of neurodevelopment.

### Transcription Factor Analysis: mRNA-TF and miR-TF Interactions

**FIGURE 7:**
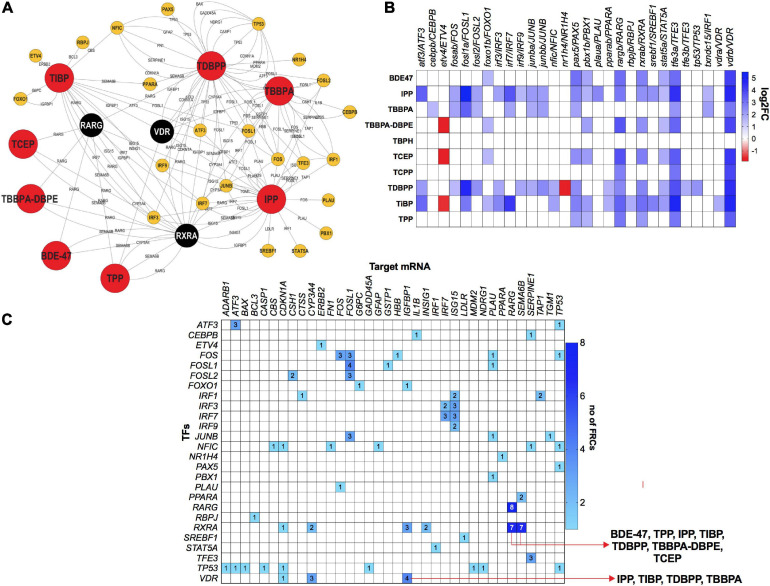
mRNA-transcription factor (TF) interactions across FRCs according to experimentally validated predictions. **(A)** FRC-mRNA-TF regulatory network based on TFmiR gene-TF interactions. 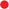 FRCs, 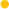 TFs. 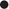 (black nodes with white text)-TFs that were differentially expressed across 5 or more FRCs, with larger nodes denoting higher number of FRCs. **(B)** Heatmap representing fold changes of selected TFs across all FRCs. Both zebrafish and human orthologs are provided. **(C)** Heatmap representing mRNA-TF combinations that were co-altered across multiple FRCs. Numbers within cells represent number of FRCs that a specific pair was altered in. Red arrows represent the FRCs for specific pairs that are discussed in the manuscript. All data based on TFmiR experimentally validated predictions.

To investigate the role of TFs in regulating mRNA and miR disruptions, we executed a TF network analysis for FRC-mRNA-TF and miR-TF interactions. Expressions of several TFs such as *VDR*, retinoic acid receptor G and retinoid X receptor (*RARG* and *RXRA*) respectively were significantly altered across multiple FRCs (Figures 7A,B) and each of these TFs were predicted to regulate several altered mRNAs (Figures 7A,C). Expressions of several mRNA-TF regulatory pairs, such as *RXR/SEMA6B* and *VDR/IGFBP1* were altered across multiple FRCs (Figure 7C).

**FIGURE 8:**
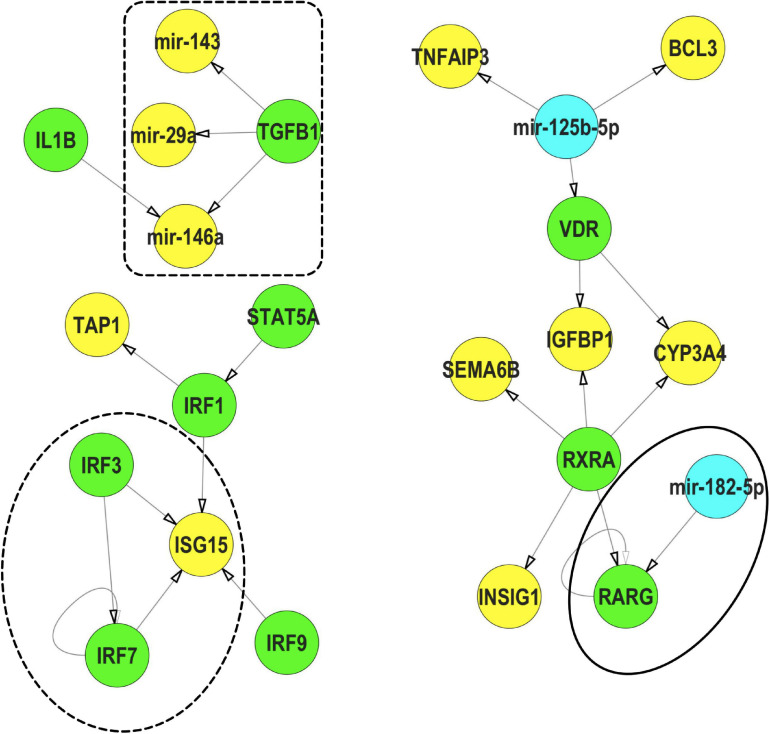
Representative interactions between mRNA, miR and TFs that show a feed forward loop (FFL, dotted circle), a TF regulating a miR (dotted box) and a miR regulating a TF (solid circle) within IPP exposures. 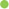 TFs, 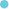 miRs, 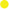 mRNA. These interactions were selected from the mRNA-miR-TF co-regulatory network for IPP. All interaction data based on TFmiR experimentally validated predictions.

We subsequently used IPP (an FRC with strong phenotypic, mRNA expression and miR expression signals) as an example to understand the potential interactions between miRs and TFs (Figure 8). Within our IPP exposures, expression of several TFs, such as *RARG* and *VDR*, were predicted to be regulated by miRs differentially expressed across multiple FRCs. Conversely, TFs such as *TGFB* and *IL1B* were predicted to regulate several miRs such as mir-146a. In addition, we also identified *IRF3/IRF7/ISG15*, a potential feed-forward-loop, in which a TF regulates another gene/TF and they both co-regulate a third gene.

### TBBPA vs TBBPA-DBPE–Impacts of Transformation on Molecular Networks

**FIGURE 9:**
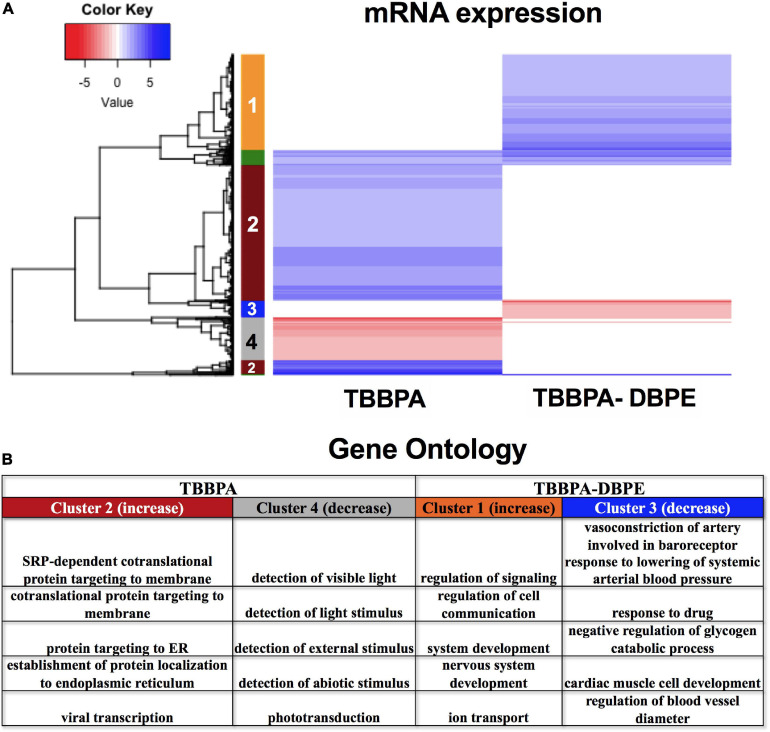
mRNA expression for TBBPA vs its derivative, TBBPA-DBPE. **(A)** Heatmap representing log_2_ fold changes of mRNA expression; colored bars on the rows represent gene clusters. Numbers within bars represent cluster numbers for specific mRNA sets. **(B)** GO processes for unique mRNAs that were increased or decreased uniquely in either FRC.

We investigated the mechanism for the differential phenotypic responses observed for the parent: derivative, TBBPA and TBBPA-DBPE. Figure 2A shows that TBBPA displayed a stronger bioactivity over its derivative as it disrupted multiple morphological endpoints. Consistent with this, TBBPA and TBBPA-DBPE displayed largely dissimilar mRNA expression patterns with minimal overlap (Figure 9A). Gene ontology analysis showed that mRNAs with unique increased (Cluster 2) and decreased (Cluster 4) expression in TBBPA were primarily associated with protein translation and light stimulus detection, respectively, while mRNAs with unique increased (Cluster 1) and decreased (Cluster 3) expression in TBBPA-DBPE were associated with drug response/vasoconstriction and system/nervous development, respectively (Figure 9B).

### Comparison of mRNA Expression Patterns of BDE-47 and Newer Generation FRCs

**FIGURE 10:**
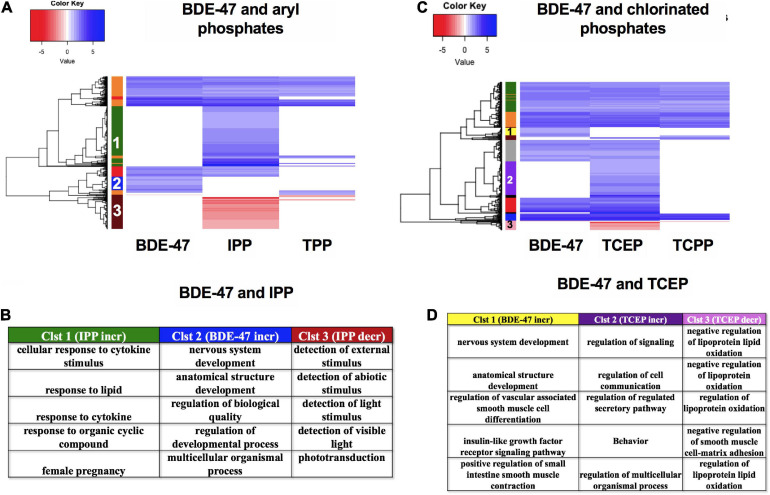
mRNA expression for BDE-47, aryl phosphates and chlorinated phosphates. **(A,C)** Heatmaps representing log_2_ fold changes of mRNA expression; colored bars on the rows represent mRNA clusters. Numbers within bars represent cluster numbers for specific mRNA sets. **(B,D)** GO processes for unique mRNAs that were increased or decreased uniquely in various clusters.

Finally, we compared the mRNA expression patterns of BDE-47, a phased out brominated flame retardant (BFR), with newer generation aryl phosphates TPP and IPP (Figures 10A,B) and chlorinated phosphates TCEP and TCPP (Figures 10C,D). mRNA clustering and GO assessments indicated that BDE-47 uniquely disrupted clusters of mRNAs associated with nervous system development and multicellular or anatomical development (Cluster 2 in Figure 10A, and Cluster 1 in Figure 10C). Comparison between the two aryl phosphates showed that IPP uniquely increased expression of mRNAs associated with lipid and cytokine response (Cluster 1) and decreased expression of mRNAs associated with responses to external stimuli (Cluster 3) (Figures 10A,B). Three mRNAs- *her4.4*, *tbx5b* and *FQ377605.1* were commonly dysregulated by both aryl phosphate OPFRCs but not any of the other FRCs. Among chlorinated phosphates, TCEP exposure uniquely increased expression of mRNAs associated with regulation of cell communication and signaling (Cluster 2) and decreased expression of mRNAs associated with regulation of lipid oxidation (Cluster 3) (Figures 10C,D). Only one mRNA (*si:ch211-188p14.2*) was common to both chlorinated OPFRCs that was not also differentially expressed in response to any other FRC.

## Discussion

### Anchoring FRC mRNA Expression to Phenotype

The primary goal of this study was to deconstruct regulatory networks for mRNA and miRs across different classes of flame retardants and identify common potential mechanisms driving toxicity. Our choice of FRCs included multiple chemical classes and bioactivity profiles, including non-responders for morphology and behavior (Figure 2A). This design allowed the investigation of exposure-driven mRNA expression landscapes, even in the absence of apparent developmental phenotypes. This is important, since many of these targets may drive adverse effects on specific organ systems or later life stages that may not be captured in developmental endpoints. Among all the FRCs tested, TBPH, a new generation brominated phthalate, elicited the least disruption of mRNA expression and no miR disruption (Figure 2B). This was consistent with previous studies where TBPH was fairly benign in *Fundulus* (Huang et al., 2019), human cell line (Xiang et al., 2017) and mouse (Chen et al., 2020) models. However, TBPH increased expression of *osbpl2a* (Figure 4A)–predicted to drive cholesterol binding and lipid transport - suggesting that, consistent with a previous zebrafish study (Guo et al., 2020), this FRC may be associated with disruption of lipid transport or metabolism. Among other FRCs, TCEP and TiBP, despite substantial transcriptomic disruption, did not produce morphological or behavioral phenotypes. This suggested that these specific mRNA expression disruptions were not linked to key developmental events, but it remains a possibility that these expression changes could manifest adverse effects at later stages.

### Neurotoxicity of FRCs

For several FRCs, nervous system development was the predominantly affected biological process (Figure 3A). Previous studies reported neurobehavioral effects of FRCs in *in vivo* or *in vitro* models, including zebrafish (Alzualde et al., 2018; Glazer et al., 2018; Truong et al., 2020), mouse (Nakajima et al., 2009) and mammalian neuronal models (Behl et al., 2015; Slotkin et al., 2017). Epidemiological studies associated prenatal organophosphate ester exposures with cognitive decline in developing children (Doherty et al., 2019). While prior studies investigated specific neurotoxic targets for individual FRCs, we, for the first time, used unbiased global experiments to report and compare common neurodevelopmental gene-sets across several classes of FRCs. We showed that several classes of FRCs altered the expression of mRNAs regulating neuronal development. Furthermore, expression of several mRNAs that modulate calcium signaling were disrupted. Calcium signaling is a crucial mechanism for several physiological processes, including neuronal electrical activity. Taken together, these suggest that disruption of neurodevelopment was a common mechanism of action for FRC-induced toxicity (Figure 3C). This was consistent with studies showing that BDE-47, chlorinated FRCs such as TCEP, TCPP and aryl FRCs such as TPP disrupted neurodevelopmental genes (Shi et al., 2018; Chen et al., 2019; Li et al., 2019). Interestingly, genetic signatures showed that neurotransmitter synthesis and activity were disrupted across several FRC classes, with the exception of chlorinated phosphates. Previous studies, using enzymatic assays, showed that among the OPFRCs, aryl phosphates, but not chlorinated phosphates, altered neurotransmitter enzymes such as acetylcholinesterase (McGee et al., 2012; Shi et al., 2018; Li et al., 2019). This corroborated with our finding that chlorinated phosphates likely do not disrupt neurotransmitter activity. The present study also suggested an epigenetically driven FRC-induced neurotoxicity since *dnmt3aa*, a regulator of methylation of transcripts that drive neuronal development (Lai et al., 2020), was overexpressed by several FRCs. Such epigenetic regulation of FRC neurotoxicity was also reflected in a previous study, which showed that TDCIPP [Tris(1,3-dichloro-2-propyl)phosphate], a chlorinated OPFRC that induced behavioral deficits in zebrafish, altered *dnmt3aa* transcript levels (Li et al., 2020). Comparison of TBBPA-DBPE and TCEP gene profiles showed that TBBPA-DBPE-induced neurobehavioral effects may be driven by a disruption of muscle structure development, an effect that may limit muscle formation and locomotor functions (Figure 6B). Based on our miR analysis, we found that expression of several miRs were commonly altered by multiple FRCs. Among these, mir-125b, mir-144, let-7 (an miR precursor), mir-9 and mir-219 were previously implicated in neuronal development, neurotoxicity and neurodegenerative diseases (Kaur et al., 2012; Novák et al., 2014). Co-regulatory analysis showed that differentially expressed mRNAs common to many FRCs were predicted to be regulated by common miRs. For example, mir-125b, whose expression was reduced by seven FRCs, was predicted to regulate a number of mRNAs that were, in turn, overexpressed by several FRCs and implicated in neurodevelopment, apoptosis and transcription (Figure 5B). Therefore, it is very likely that a single miR can impact a multitude of biological processes. Comparisons between TBBPA-DBPE and TCEP demonstrated that TBBPA-DBPE-induced dysregulation of *WNT3A*, a member of Wnt pathway known to impact neurodevelopment and neurodevelopmental disorders such as autism (Kumar et al., 2019). *WNT3A* mRNA interacted here with mir-15a-5p, again suggesting that key miRs may be involved in driving FRC-induced neurotoxicity (Figure 6C).

### Lipid Metabolism and Other FRC-Disrupted Biological Processes

Lipid response was disrupted by exposures to non-halogenated OPFRCs, corroborating with previous reports of disrupted lipid metabolism/transport resulting in facilitated adipogenesis and disruption of liver development and function (Du et al., 2016; Cano-Sancho et al., 2017; Reddam et al., 2019). mir-144 and mir-92a regulate gene expression driving lipid metabolism (Aryal et al., 2017). Indeed, previous studies revealed that miR-92a binds to the 3′UTR of *OSBPL2*, a gene that regulates lipid binding and transport (Helwak et al., 2013), and this miR was impacted by several OPFRCs in our dataset (Figure 5B). This suggests that disruption of lipid-related processes may be regulated by miRs. Differentially expressed mRNAs with several other key functions, such as transcription, skeletal development and angiogenesis were also predicted to be regulated by key miRs, indicating that miRs play an important role in overall developmental responses to FRCs.

TBBPA was the only FRC that produced widespread mRNA expression changes where the nervous system was not the primary target. This was consistent with the observation that TBBPA did not alter zebrafish behavior and that the nervous system was not a direct target of this FRC as reviewed in Kacew and Hayes (2020). Instead, TBBPA primarily impacted pathways that affect synthesis and targeting of proteins that enter the endoplasmic reticulum, as well as translation, which may explain the significant morphological defects observed. It is important to note that TBBPA is primarily a reactive FRC (chemically bound to product matrix) and likely has a lower potential to leach into the environment compared to additive (not chemically bound to product matrix) FRCs.

### mRNA-miR and mRNA-TF Interactions-Carcinogenic Signature

Zebrafish has been widely used as a model for studying cancer mechanisms (Hason and Bartůněk, 2019). Indeed, zebrafish developmental toxicity assays can detect carcinogens with ∼80% concordance with rodent data (unpublished data). miRs are important in tumorigenesis and our mRNA-miR interactions revealed FRC carcinogenic signatures. mir-125b is a known onco-miR that acts as a tumor suppressor; downregulation of this miR has been shown in several types of cancerous tissues (Wang et al., 2020). In our data, specifically for OPFRCs, reduced expression of mir-125b was associated with increased expression of *BCL2* and *TP53* (anti-apoptotic genes), *ERBB2* (an androgenic gene) as well as *CYP24A1* and *VDR* (Figure 5B); negative correlations of mir-125b with all of the aforementioned genes have been previously evidenced in several cancer types (Mohri et al., 2009; Banzhaf-Strathmann and Edbauer, 2014; Wang et al., 2020). Our mRNA-miR interaction data suggests the need to confirm and further understand the carcinogenic potential of current and emerging FRCs.

Expressions of several TFs such as *RARG*, *RXR* and, all known for their role in carcinogenesis, were frequently altered by several FRCs (Figures 7A,B). *RAR* and *RXR* are nuclear receptor transcription factors; RXRs heterodimerize with RARs and VDR and play important roles in regulation of genes that control cell proliferation (Dawson and Xia, 2012), specifically in tumor or cancer cells. Indeed, *RAR-RXR* dimerization has been targeted as a therapeutic option in acute promyelocytic leukemia using retinoids or rexinoids (Altucci et al., 2007). Here, the altered expression of both *RAR* and *RXR* was common to 7 FRCs (BDE-47, TPP, IPP, TiBP, TDBPP, TBBPA-DBPE, and TCEP) suggesting that there may be a common operant mode of action (Figure 7C). *RXR*s also heterodimerize with *PPAR*s to regulate the expression of *SEMA6B*, a gene highly expressed in breast cancer (Murad et al., 2006). We observed disruption of both *RXR* and *SEMA6B* mRNA levels by the same 7 FRCs, and among these, *PPARA* was differentially expressed by TiBP and TDBPP. Likewise, *RXR/RXR* and *VDR/RXR* heterodimers also regulate the expression of *IGFBP1* (Baxter, 2014), a tumor suppressor gene that was differentially expressed in at least 4 FRCs (IPP, TiBP, TDBPP, TBBPA). These data show that, similar to mRNA-miR interactions, FRCs may drive altered responses in mRNA-TF co-regulations that are carcinogenic signatures.

It is important to note that many these carcinogenic genes and TFs are not exclusive to cancer incidence, but also have crucial roles in development, often in their target tissues (Naxerova et al., 2008). For example, although *RARs* and *RXRs* are known for their roles in cancer, they also play an important role in development, specifically cardiac development (Rhinn and Dollé, 2012). In fact, previous zebrafish studies illustrate that *RAR* and *RXR* may be involved in cardiotoxicity induced by in TPP (Isales et al., 2015; Mitchell et al., 2018) and monosubstituted isopropylated triaryl phosphate, an organophosphate component of the flame retardant mixture Firemaster 550 (Haggard et al., 2017), respectively. This facet holds true for miRs as well; for example, mir-125b plays an important role in early neural specification and neurodevelopment (Boissart et al., 2012). Such studies often lack prominence since the majority of the validations are done in cancer models. Our data highlights the need for a better investigation of FRC-disrupted mRNA-TF and mRNA-miR regulations for their toxicological roles in in the context of development.

### Interaction Between miR-TFs and Feed-Forward-Loop in IPP Exposures

Using IPP as an example, we showed specific examples of less common miR-TF interactions (Figure 8). For example, among the differentially expressed transcripts, *IL1B* interacted with mir-146a, an important pattern in inflammatory stress responses (Nahid et al., 2015). Conversely, mir-182 interacted with the TF *RARG*, a co-regulation signature of stress-induced senescence in human fibroid cell lines (Li et al., 2009). We also demonstrated an example of a feed-forward-loop (FFL)– a three-gene-pattern where one TF (*IRF3*) regulated the expression of a second TF (*IRF7*) and they both regulated the expression of a gene (*ISG15*) with an important role in innate immunity (Perng and Lenschow, 2018), suggesting that such networks may be commonly operant in response to FRC exposures. Study of FFLs are important since they accelerate expression of specific genes (Jin, 2013) and have been implicated in several diseases, including neurodegenerative diseases (Albeely et al., 2018). Our data suggested that such intricate molecular co-modulatory dynamics may drive FRC health effects and should be investigated.

### TBBPA vs TBBPA-DBPE–Role of Transformation

TBBPA-DBPE was chosen as a derivative and transformative product of TBBPA and both of these are used as FRCs in polymers (Knudsen et al., 2007; Qu et al., 2013). While TBBPA can induce oxidative stress and neurobehavioral deficits (Chen et al., 2016; Wu et al., 2016), our data showed that the predominant effect was likely disruption of protein localization and disruption of translation resulting in large-scale morphological effects. For TBBPA-DBPE, neurodevelopment was an important targeted gene process (Figure 9), consistent with its behavioral phenotype. Though closely related structurally, gene expression changes following TBBPA-DBPE exposures were significantly different from TBBPA, with minimal overlap in gene expression, miRs and TFs.

### Comparison Between BDE-47 and Replacement OPFRCs—Are They a Safer Alternative?

We also compared the mRNA expression signatures of BDE-47 with that of aryl and chlorinated OPFRCs (Figure 10). This was important to address whether the replacement of BDEs by OPFRCs is, in general, a safer alternative. Both our phenotypic and molecular data showed that, consistent with previous BDE-47 behavioral data (Chen et al., 2014), nervous system development was a key target unique to BDE-47. But the replacement OPFRC, IPP, was more bioactive, with cytokine responses potentially being a key mechanism of its toxicity. Among the chlorinated OPFRCs, consistent with previous work (McGee et al., 2012), neither TCEP nor TCPP produced phenotypic disruptions; however, TCEP produced widespread gene expression changes with cell signaling and lipid regulation being the most disrupted processes. Ours and other studies (Blum et al., 2019), would suggest that a blanket conclusion of greater safety with OPFRCs is not warranted, but given their greater structural diversity, safer chemistries among the existing OPFRs can be identified and used to guide incorporation of moieties for even greater safety.

## Conclusion

Prior efforts to group the toxicity of FRCs by chemical class proved challenging. A more nuanced and sensitive approach was required. Since human exposure to FRCs remains a major public health concern, this study represents a significant advance by anchoring whole animal developmental phenotypes to their underlying mRNA-miR expression responses. Our results illustrate that while there are commonalities in mRNA and miR expression changes across different FRC physicochemical classes, FRCs belonging to identical classes can induce starkly different gene expression profiles and may need to be evaluated individually. Through computational analysis, we propose, for the first-time, predictive dynamics between miRs and mRNA targets that together may help to develop complex adverse outcome pathways for FRCs. Coupling phenotypic outcomes to gene expression in zebrafish offers rapid and powerful opportunities to guide the selection of safer flame retardant replacements for commercially important products. Finally, our study also demonstrates that miRs are key to understanding toxicological mechanisms and such regulatory network assessments between mRNA and miRs should be important considerations in toxicological profiling.

## Data Availability Statement

The data were deposited to NCBI under the GEO accession number GSE169013.

## Ethics Statement

The animal study was reviewed and approved by Institutional Animal Care and Use Committee.

## Author Contributions

LT, MS, and RT contributed to conception and design of the study. SD, CD, and CS organized the database. SD and CD performed the statistical analysis. SD analyzed and interpreted the data, made figures and wrote the first draft of the manuscript. All authors contributed to manuscript revision, read, and approved the submitted version.

## Conflict of Interest

The authors declare that the research was conducted in the absence of any commercial or financial relationships that could be construed as a potential conflict of interest.

## Footnotes

1
https://service.bioinformatik.uni-saarland.de/tfmir/

2 http://cytoscape.org
