# Supplementary material for: Phenotypically Anchored mRNA and miRNA Expression Profiling in Zebrafish Reveals Flame Retardant Chemical Toxicity Networks
Source: Front Cell Dev Biol. 2021 Apr 9;9:663032. doi: 10.3389/fcell.2021.663032 (PMC8063052; doi:10.3389/fcell.2021.663032)
Supplement: Supplementary file 4 [file Data_Sheet_1.DOCX]

Phenotypically anchored mRNA and miRNA expression profiling in zebrafish reveals flame retardant chemical toxicity networks

Subham Dasgupta^1^, Cheryl L. Dunham^1^, Lisa Truong^1^, Michael T. Simonich^1^, Robyn L. Tanguay^1^*

^1^Department of Environmental and Molecular Toxicology, Sinnhuber Aquatic Research Laboratory, Oregon State University, Corvallis, Oregon, USA

Supplemental Tables- 3

Supplemental Figures- 1

**Supplemental table legends:**

**Table S1**- Phenotypic, behavioral, mRNA and miR data for the selected FRCs. Phenotyping and behavioral data represented as lowest effect levels (LELs, Table S1.1). mRNA and miR data represented as log_2_ fold changes (Tables S1.2 and S1.3). Blank cells represent fold changes below statistical or fold change thresholds.

**Table S2-** TFmiR miR-TF-miR interaction data for all FRCs. Only experimentally validated data (Table S2.1), both experimentally validated and computationally predicted data (Table S2.2-2.9)

**Table S3**. Gene Ontology data for mRNA (Tables S3.1-3.10) and mRNA targets of miRs (Table S3.11)

**Supplemental Figure legends**

**Figure S1.** Heatmap representing log_2_ fold changes of (A) all differentially expressed mRNAs and (B) all differentially expressed miRs across FRCs.

**Figure S1**
